# Prenatal sex determination in suspicious cases of X-linked recessive diseases by the amelogenin gene

**Published:** 2014-02

**Authors:** Amir Abbas Rahimi, Mohammad Hassan Shahhosseiny, Ghasem Ahangari, Jalal Izadi Mobarakeh

**Affiliations:** 1 Molecular Medicine Department, Pasteur Institute of Iran, Tehran, Iran; 2 Department of Microbiology, Islamic Azad University, Shahr-e- Qods Branch, Tehran, Iran; 3 Medical Biotechnology Department, National Institute of Genetic Engineering and Biotechnology, Tehran, Iran; 4 Department of Pharmacology, Tehran Medical Science Branch, Islamic Azad University, Tehran, Iran

**Keywords:** Amelogenin gene, Prenatal diagnosis, Sex determination, X-linked diseases

## Abstract

***Objective(s):*** To determine the fetal discernment in suspected cases of sex linked recessive disease in the first trimester of pregnancy.

***Materials and Methods:*** After collection of 100 Chorionic Villi samples, the DNAs were extracted and baby gender was determined. Meanwhile, after increasing the sensitivity, the system was able to detect the sex of each cell which was obtained by biopsy**.**

***Results:*** Early fetal gender of 100 Chorionic Villi samples were assessed by PCR. After increasing sensitivity of the assay, the sexes in 13 fetuses that were in different cellular stages were detected. Morover, sexes were detected in two unfertilized and one fertilized ovum but without any division.

***Conclusion:*** Sex detection of fetus before delivery in the first trimester of pregnancy, will prevent babies with abnormalities being born. It can also be used in detection of recessive sex related diseases in *In Vitro* Fertilization cases for sex detection and to transfer female fetus to the mother. Our optimized molecular detection system was designed on the basis of amelogenin gene, which can determine the sex in blood, chorionic villi, and single cell *in vitro* fertilization with high sensitivity and specificity.

## Introduction

Sex determination has several applications in biological sciences, especially in medicine (1). More-over, the most important application of sex determ-ination in medicine is in prediction of X-linked genetic diseases (2), because carrier mothers have 25% chance to give birth to a baby with related abnormalities, such as Hemophilia and Duchenne Muscular Dystrophy (DMD) (3). There are different of methods for sex determination (4, 5) such as Chorionic Villus Sampling (CVS) technique for obtaining genetic information from fetus in the first trimester (6).

Molecular techniques such as polymerase chain reaction (PCR) provide sensitive and rapid appro-aches for the analysis and detection of genes in each DNA. Our target gene is the amelogenin gene (AMEL). The AMELX gene has a size of 2872 bp and is located on the p22 region of the X-chromosome, while the AMELY gene has a size of 3272 bp and is located on the 11p12.2 region of the Y-chromosome (7). Sex identification by the amelogenin test is based on the detection of different sizes in the amplified products from the two sexual chromosomes.

In this research we aimed to describe a method for sex determination in the first trimester, using Chorionic Villi (CV) and to increase the sensitivity of the system for one cell sex identification of biopsy samples from embryo in 4-8 cell stage in suspected families of X-linked recessive diseases.

## Methods and Materials

***Chorionic Villus Sampling***

**Figure1:**
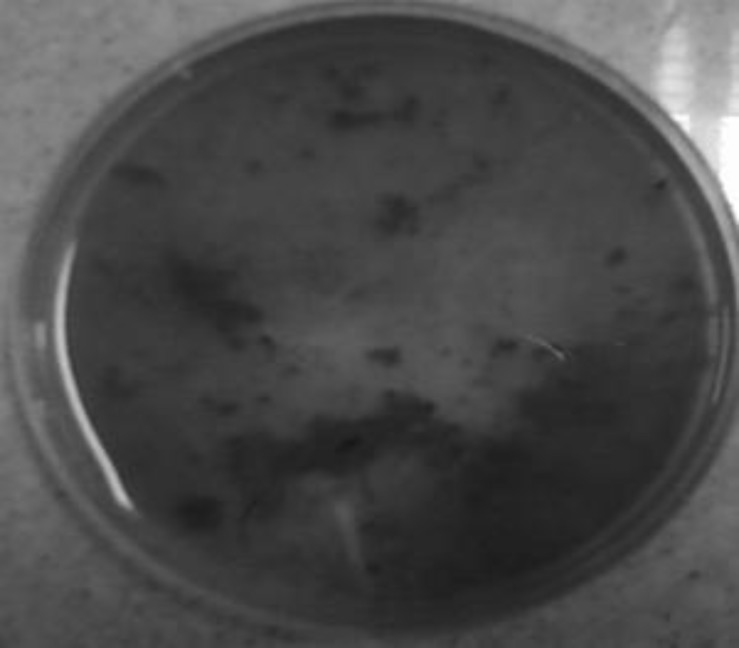
Chorionic villi samples for sex determination

One hundred CV samples were collected in sterile Hank's balanced salt solution (Sigma) from pregnant women in their 9-14 weeks of gestation under the ultrasound guidance from Novin Medical Center in Tehran, Iran. In this study, CV samples were separated from maternal deciduous tissue by dissection or separation, using fine forceps under inverted microscope (Zeiss, Germany). It was important that the CV of fetal not being contaminated with maternal samples (Figure1). 

Then, 10-30 mg of CV was added to a sterile 1.5 ml Eppendorf microtube for DNA extraction.

***Extracting DNA n from CVS***

Initially, 300 µl Trypsin (0.5%, Sigma) was added to each of the tubes (each one containing 10-30 mg of CV), and shaken vigorously for 2 min (IKA, Germany). Furthermore, slices of CV were precipit-ated by centrifugation (Eppendorf, Germany) at 8000 rpm for 1 min. Then, trypsin was discarded, and 100 µl of NaOH (50 mM, Sigma) was added. After mixing, tubes were put in boiling water (100°C) for 20 min (Clifton, England). In the next step, 20 µl Tris-HCl with pH=7.5 was added to each tube and the samples were centrifuged for 1 min at 10000 rpm (Eppen-dorf, Germany). Finally, the supernatant was transferred to a new tube.

***Polymerase Chain Reaction***

Primers described by Nakahori *et al* (1991) were synthesized by Fermentas Co. (Fermentas, Lithuania) and used for amplification of CV DNA (8). Sequences of two pairs of primers (outer and inner) are: outer 5'-CTGATGGTTGGCTCAAGCCTGTG-3', 5'-TAAAGAGATTCATTAACTTGACTG-3' and inner 5'-TCCTCCTAAATATGGC(T/C)GTA-3', 5'- AGAAAA (C/T)C TTGCCTCA(G/A)A(T/A)T-3'.

Nested PCR was performed with the CV DNA (20-50 ng/µl, 5 µl) in a 25 µl master mix that contained 2.5 µl 10X PCR buffer, 0.75 µl MgCl_2_ 50 mM, 0.5 µl dNTPs 10 mM (Fermentas, Lithuania), 0.2 µl Taq DNA polymerase (1 unit) (Fermentas, Lithuania), 1.25 µl forward and 1.25 µl reverse 10 pmol primers (outer), in the first round. The second round was similar to the first round; however, only forward and reverse inner primers were used. Amplification was performed on Mastercycler Gradiant thermocycler (Eppendorf, Germany).

The first round PCR amplification was carried out by denaturation of the template at 94°C for 3 min, followed by 30 cycles of incubation at 94°C for 1 min, and 56°C for 40 sec, 72°C for 1 min, and finally, incubation at 72°C for 5 minutes. Then, 100-fold-diluted PCR product was used from the first round as template for the next round. The second round of amplification was performed by denaturation at 94°C for 1 min, 48°C for 40 sec, 72°C for 40 sec, and final incubation at 72°C for 5 min. Then, the second round PCR products were analyzed on a 2% TopVision^TM^ Agarose gel (Fermentas, Lithuania) by using TBE (90 mM Tris-HCl, 90 mM boric acid and 2 mM Na_2_EDTA, Sigma), stained by ethidium bromide (0.5 µg/ml, Sigma), and were evaluated, using ultraviolet light (9).

***Increasing sensitivity of the system***

First, the DNA from leukocytes of peripheral venous blood samples was extracted by standard procedures (9). Subsequently, the amount of isolated DNA was quantitated by ultraviolet spectrophoto-metry technique (Optima, Japan). It was established that an OD=1 corresponds to 50 µg/ml of double strand DNA (11). The extracted DNA was diluted up in TE buffer pH=7.4 (Tris-HCl 10 mM and EDTA 1 mM, Sigma) until it reached 5-10 pg of DNA which corresponds to reaching one human cell (1). At the end, the number of cycles in the second round was increased up to 40 cycles for amplification one cell.

***Biopsy and DNA extraction from one cell***

Single cells were taken from the Center of Non-Fertility and Sexual Disability of Kosar, Tehran. Blastomere biopsy was performed in the morning of the third day, after *in vitro* fertilization (IVF) by a pair of micromanipulator (Narishige, Japan) in conjunction with an inverted microscope (Olympus, Japan), when embryo normally reaches 4-8 cell stage. In the next step, single cell was transferred to a sterile microtube that was composed of lysis buffers (200 mM KOH and 50 mM DTT, Sigma).

## Results

**Figure 2:**
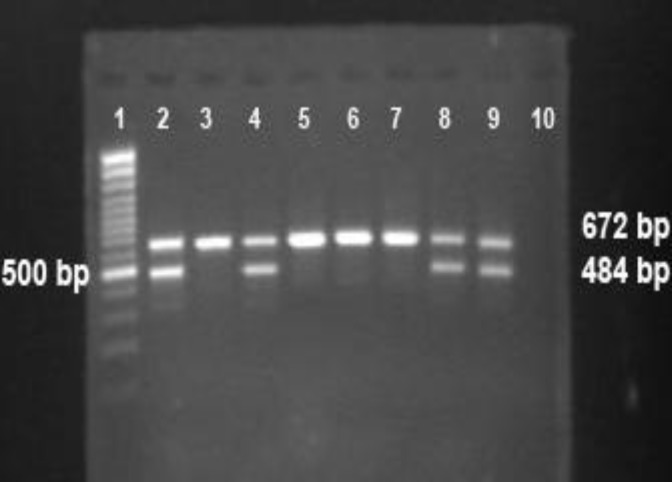
PCR analysis on DNA extracted from CVS samples. Lane 1 is 100 bp size marker (Fermentas, Lithuania); lane 2, 4, 8 and 9 are male and lane 3, 5, 6 and 7 are female and lane 10 is negative control

Among the 100 C.V samples obtained from pregnant women in their 9-14 weeks, 44 were predicted to be male and fifty six to be female. The results of the CV DNA's amelogenin gene analysis in some individuals are demonstrated in Figure 2. In the male cases, two bands (484 and 672 bp) were visible, whereas single 672 bp band was present female. The presence of the 484 bp band represent-ting the specific sequence of Y-chromosome, plus the 672 bp band which represent the specific sequence of X-chromosome in the CV sample is an indicative of male fetus. However, the presence of only 672 bp band in an individual CV sample reveals a female fetus. Without band of 672 bp, the fetal sex determination would be unreliable. The difference between two band sizes (X and Y-chromosome) arises from deletion of 189 in the third intron of Y specific Amelogenin.

## Discussion

X-linked recessive diseases occur with an estimated incidence of about 0.5%, and often result in severe phenotypes (10). Male offspring of women who are carriers an X-linked disorder have a 50% chance of inheriting the condition. Female offspring will not, in most conditions, manifest the disorder; however, they have a 50% risk of being carrier like their mother. Therefore, carrier mothers of serious X-linked conditions such as Duchenne Muscular Dystrophy, Hunter’s disease, Haemophilia, Lesh-Nyhan syndrome and Adrenoleucodystrophy, which confer significant morbidity or mortality, therefore, require genetic prenatal diagnosis only when the fetus is male (11). 

Diagnostic testing such as Chorionic Villus Sampling (CVS), for many of these conditions is available. This technique is the earliest procedure for fetal sex determination and molecular analysis of X-linked genetic disorders in the first trimester of gestation, compared with the amniocentesis in the second trimester (3, 12). Cordocentesis is another less commonly used method, which is usually done after eighteen weeks, and its total loss rates are significant. CVS allows evaluation fetus through aspiration a portion of the developing placenta using a transcervical or transabdominal approach. CVS is usually performed at 9-14 weeks. This technique allows the option of earlier abortion in the case of any fetal anomalies (12). The benefits of CVS mostly arise from the gestation at which it can be performed. This allows early diagnosis; will undoub-tedly reduce the level of stress in the parents waiting for a diagnosis, and enables them to have the option of surgical termination of pregnancy (3).

Molecular techniques such as PCR provide sensitive and rapid approaches for the analysis and detection genes in each DNA and even one cell (5-10 pg of DNA). The amelogenin gene can be used for sex determination. These genes are found on both X and Y-chromosomes and have significant sequence similarity (7). There is difference between the two chromosomes in term of size (the main difference is the deletion of 189 bp in third intron of Y-chromosome) (8), which can be used to differentiate males from females. The major advantage of using this gene is that it can be amplified by one set of primers that target the same region on both the X and Y-amelogenin gene. Another advantage of this system is having internal control. It means X-chrom-osome specific band shows success of the test (acts as internal control) and presence Y-chromosome specific band shows sex of the sample.

Smid *et al*, (1999) have found the sensitivity of 100% in fetal sex assignment, using a conventional nested PCR on plasma DNA (12). This alternative PCR technique had high sensitivity and specificity. The author and his colleagues could amplify genomic DNA one cell (5-10 pg) after increasing the second round to 40 cycles. They were able to detect the sex from one cell after biopsy of blastomere from embryo in 4-8 cell stage. Some researchers did sex identification by amplifying the sequence on Y-chromosome (13, 14), whereas, due to failure in amplification, wrong result was reported. In addition, many studies just used sequences related to Y-chromosome for sexing (15) that were unreliable, because lack of any PCR products (no amplification) is interpreted as female. Failure of amplification can be arisen from materials used in amplification or operator, in some cases as a result of inhibitor, especially sex determination in remains of corpse.

Preimplantation genetic diagnosis (PGD) was originally developed as an alternative to prenatal diagnosis to reduce the transmission of severe genetic diseases form fertile couples with a repr-oductive risk (2). PGD is a technique that utilizes molecular techniques to analyze single cells removed from preimplantation embryos within early cleavage stages. The first clinical application of PGD by Y-chromosome-specific sequences was performed using PCR for determination the sex of the embryos obtained from couples at risk X-linked disease. Couples at risk of having an affected child can have embryo diagnosis before implantation, thereby avoiding the termination of an affected pregnancy (16). Several religions, including Judaism and Islam, are against abortion at later stages of pregnancy; however, they have no objection to perform PGD, because it is believed that a pregnancy is only established when the mother can detect fetal movement (17).

## Conclusion

Chorionic Villus Sampling and Nested PCR are the important techniques for fetal sex determination and molecular analysis of X-linked genetic disorders in the first trimester of gestation in the compared with other techniques. Those allow the option of earlier detection and abortion in the case of any fetal anomalies. The amelogenin gene can be used for sex determination. These genes are found on both X and Y-chromosomes. The advantage of this system is having internal control. As a result, we can amplify target gene with one set of primer in each round.
